# Emergency high ligation in a suspected COVID-19 pediatric patient with incarcerated inguinal hernia: A case report

**DOI:** 10.1016/j.amsu.2021.01.075

**Published:** 2021-01-25

**Authors:** Gunadi, Munawir Makkadafi, Aditya Rifqi Fauzi, Amsyar Praja, Kemala Athollah, Marcellus

**Affiliations:** a Pediatric Surgery Division, Department of Surgery, Faculty of Medicine, Public Health and Nursing, Universitas Gadjah Mada/Dr. Sardjito Hospital, Yogyakarta, 55281, Indonesia; b Pediatric Surgery Division, Department of Surgery, Faculty of Medicine, Public Health and Nursing, Universitas Gadjah Mada, Yogyakarta, 55281, Indonesia; c Department of Surgery, Faculty of Medicine, Hasanudin University, Makassar, 90241, Indonesia; d Undata Distric Hospital, Palu, 94116, Indonesia

**Keywords:** COVID-19, Emergency high ligation, Incarcerated inguinal hernia, Pediatric case, SARS-Cov-2, Tertiary protection regulations

## Abstract

**Background:**

SARS-Cov-2 infects not only adults, but also children, including pediatric surgery patients with acute abdomen. Here, we report a pediatric surgery case with incarcerated inguinal hernia and suspected COVID-19.

**Case presentation:**

A 11-month-old male was brought to our emergency department with the main complaint of recurrent yellowish-green vomiting that was experienced from one day before admission. High fever and shortness of breath were also reported. This patient was also suffering from moderate dehydration. Neither history of contact with a confirmed case of COVID-19 nor traveling from any local transmission area were found. However, a SARS-CoV-2 rapid antibody test revealed a positive result. A lump in the left scrotum that persisted during admission was found. Fluid resuscitation and nasogastric tube placement for decompression was performed. Manual reduction was attempted but failed to reduce the lump. Accordingly, we decided to perform an emergency high ligation using tertiary protection regulations, *i.e.,* full personal protective equipment (PPE) for COVID-19. Intraoperatively, we found a small intestine loop trapped in the scrotum and stuck in the inguinal canal. Postoperatively, the baby was continued to be managed as a patient with COVID-19 while waiting for the real-time reverse transcription polymerase chain reaction (RT-PCR) results.

**Discussion:**

Manual reduction is standard treatment for incarcerated inguinal hernia in children. The successful rate of manual reduction is about 70%, therefore, if the manual reduction fails, an emergency surgery is mandatory.

During the COVID-19 pandemic, all medical procedures require clarity of the patient's status including whether infected with COVID-19. Along with proper precautions, great care must be taken during surgery to minimize the risk of cross infection to health workers.

**Conclusions:**

During the COVID-19 pandemic, surgeons should always be aware of the possibility of cross-transmission from the patient, since children are also susceptible to SARS-CoV-2 infection. When and wherever possible, surgeons should perform the procedure in the quickest and most effective manner to shorten exposure time with patient and anesthetic aerosols as well as using appropriate PPE.

## Highlights

• Manual reduction is standard treatment for incarcerated inguinal hernia in children.
• Children are also susceptible to SARS-CoV-2 infection.
• Surgeons should perform the procedure in the most effective manner during pandemic.

## 1 Introduction

The ongoing outbreak of Coronavirus Disease 2019 (COVID-19), which is caused by Severe Acute Respiratory Syndrome Coronavirus 2 (SARS-CoV-2), has spread throughout the world. Until January 19, 2021 this global pandemic has infected more than 96 million people in over 220 countries/territories and caused 2,063,299 deaths [1]. Rapid transmission of the virus can make hospitals overwhelmed, causing non-urgent operations to be delayed or even canceled [2]. This change of surgery scheduling is important in order to increase hospital bed capacity and maintain the availability of sufficient personal protective equipment (PPE) for health workers [3].

However, at the same time, pediatric surgery services have become severely affected [4]. For patients who experience life-threatening conditions, although surgical procedures in children with SARS-CoV-2 infection can pose risks of cross-transmission, the choice of an emergency operation may be the only lifesaving method when conservative treatment fails [5]. Here, we report a pediatric surgery case with incarcerated inguinal hernia and suspected COVID-19. This study has been reported in line with the SCARE guidelines [6].

## 2 Presentation of case

**Fig. 1:**
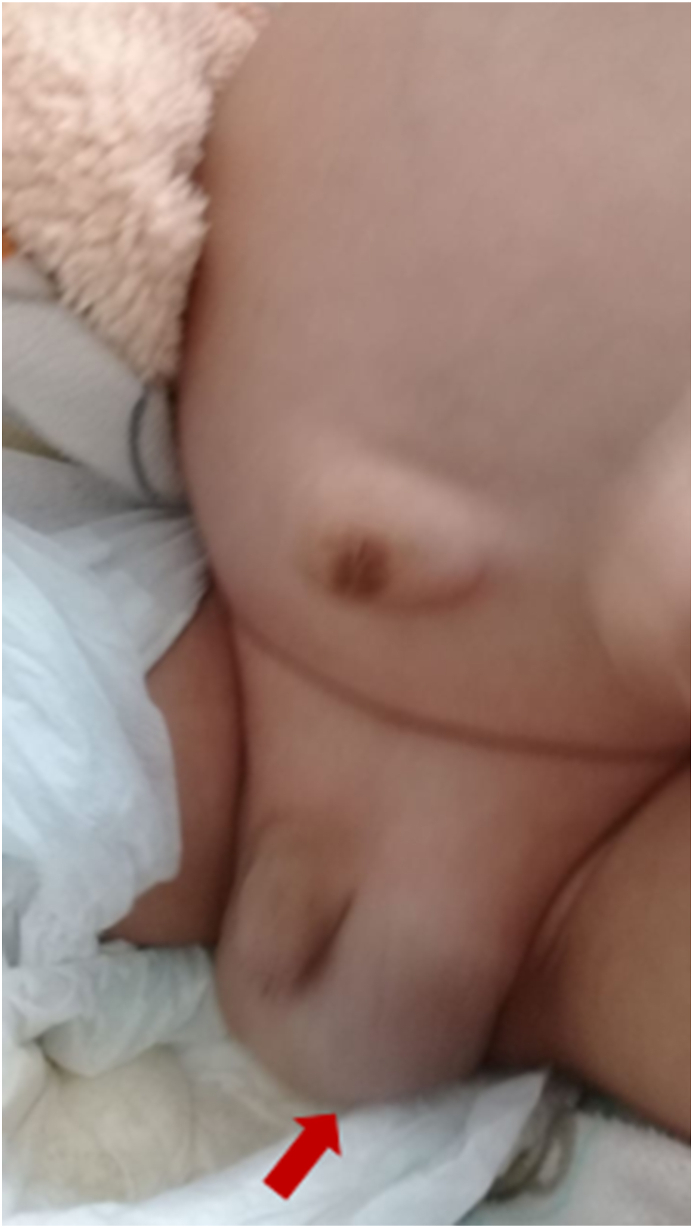
A lump was noted in the left scrotum.

**Fig. 2:**
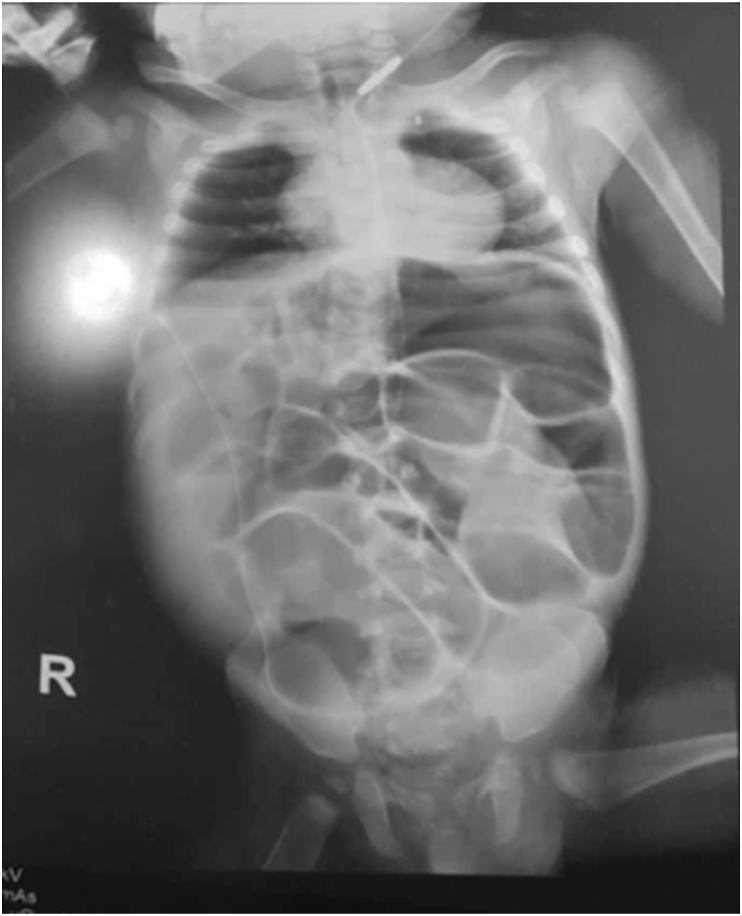
Babygram showed dilated bowel loops, suggesting ileus.

An 11-month-old male was brought to our emergency department with the main complaint of recurrent yellowish-green vomiting that was experienced from one day before admission. The patient's abdomen was bloated, while the last defecation was the day before coming to the hospital. High fever and shortness of breath were also reported. On physical examination, his general condition was lethargic, and body weight was 7 kg. Vital signs revealed pulse of 140 times per minute, respiratory rate 40 times per minute, body temperature of 39.5 °C, and capillary refill time <2 seconds. In the head examination, a sunken fontanel and eyes were found, indicating moderate dehydration. His abdominal wall was distended, and auscultation revealed an increase in bowel sounds, without any abdominal tenderness. A lump in the left scrotum that persisted during admission was found (Fig. 1). The lump had come and gone for 10 days. Routine laboratory findings showed leukocytosis, while the babygram revealed dilated bowel loops which suggested ileus (Fig. 2). Neither history of contact with a confirmed Case of COVID-19 nor traveling from any local transmission area were found. However, a SARS-CoV-2 rapid antibody test revealed a positive result. Fluid resuscitation and nasogastric tube placement for decompression were performed. Manual reduction was attempted but failed to reduce the lump.

Accordingly, we decided to perform an emergency high ligation using tertiary protection regulations, *i.e*., with full PPE for COVID-19. Intraoperatively, we found a small intestine loop trapped in the scrotum and stuck in the inguinal canal. Neither necrosis nor perforation were found in the intestinal loop. The intestine was returned into the peritoneal cavity followed by a high ligation. Postoperatively, the baby was continued to be managed as a patient with COVID-19 while waiting for the real-time reverse transcription polymerase chain reaction (RT-PCR) results.

## 3 Discussion

Manual reduction is standard treatment for incarcerated inguinal hernia in children. Moreover, this technique is very useful to decrease the need for any emergency procedure during the COVID-19 pandemic [7]. However, manual reduction is not without some risks. Several complications may occur following manual reduction, including peritonitis and bowel rupture [8]. The successful rate of manual reduction is about 70%, therefore, if the manual reduction fails, an emergency surgery is mandatory [8].

During the COVID-19 pandemic, all medical procedures require clarity of the patient's status including whether infected with COVID-19. Along with proper precautions, great care must be taken during surgery to minimize the risk of cross infection to health workers. This includes PPE and control of aerosols or body fluids produced during operations that might be contaminated [9]. Besides the thoracic X-rays, preoperative COVID-19 screening using rapid antibody tests has become a protocol in the surgery department of our hospital, particularly for emergency patients. Our patient showed a positive result of the rapid antibody test for COVID-19. In our institution, the RT-PCR results will be obtained four days after the swab samples are taken. However, in this case, the surgical time window was limited and the patient deteriorated after failure of manual reduction. Accordingly, we decided to perform an emergency high ligation using tertiary protection regulations, *i.e.,* with full PPE for COVID-19, while waiting for the RT-PCR results. The final RT-PCR results of the patient were negative for two consecutive swab tests. In spite of this, there is a possibility of false negative results of RT-PCR in patients with COVID-19 [10].

High fever in our patient could be caused by ileus complications due to fluid sequestration to the third cavity which results in dehydration and may be accompanied by bacterial translocation [11]. It might also be caused by the body's reaction to the SARS-CoV-2 infection [[12], [13], [14]]. In addition, shortness of breath in our patient might be caused by abdominal distension due to bowel dilatation [15] or by SARS-CoV-2 infection of the lungs [16].

The use of operating rooms specifically for patients with COVID-19 which are separated from non-COVID-19 patients is also one way to reduce the risk of nosocomial infections [17]. In our hospital, we have prepared a containment area specifically designed as an emergency operating room according to strict patient and personnel safety standards. We use separate pathways for the patient and health workers to enter the operating theater, and our operating room is also equipped with negative pressure. These measures are in accordance with recommendations from Liu et al. [18].

The surgical procedures for open high ligation in children before and during the COVID-19 pandemic are the same. However, considering the possibility of the surgeons' and other surgical team members’ exposure time to anesthetic aerosols and body fluids that might be contaminated with the SARS-Cov-2, it is recommended to perform the operation in the quickest and most efficient manner [18]. Furthermore, post-operative follow-up should strictly obey the tertiary protocol regulations by using full PPE [19].

Notably, while the diagnosis of COVID-19 is according to RT-PCR, there are several obstacles of detection of SARS-CoV-2 in developing countries, such as the PCR reagents supply, the qualified laboratory staffs and the laboratories with appropriate biocontainment levels accessibility [20,21]. Therefore, a few institutions still use the rapid antibody test for COVID-19 screening while waiting for the RT-PCR results, especially for emergency surgical patients.

## 4 Conclusions

During the COVID-19 pandemic, surgeons should always be aware of the risk of cross-transmission from the patient, since children are also susceptible to SARS-CoV-2 infection. When and wherever possible surgeons should perform the procedure in the quickest and most effective manner to shorten exposure time with the patient and anesthetic aerosols as well as using appropriate PPE.

## Sources of funding

This study was funded by 10.13039/501100009509Indonesian Ministry of Research and Technology/National Agency for Research and Innovation.

## Ethical approval

The informed consent form was declared that patient data or samples will be used for educational or research purposes. Our institutional review board also do not provide an ethical approval in the form of case report.

## Consent

Written informed consent was obtained from the parents’ patient for publication of this Case report and accompanying images. A copy of the written consent is available for review by the Editor-in-Chief of this journal on request.

## Author contribution

Gunadi, Munawir Makkadafi and Amsyar Praja conceived the study and approved the final draft. Gunadi and Aditya Rifqi Fauzi drafted the manuscript. Kemala Athollah and Marcellus critically revised the manuscript for important intellectual content. All authors read and approved the final draft. All authors facilitated all project-related tasks.

## Research registration

The manuscript is a case report, not considered a formal research involving participants.

## Guarantor

Gunadi.

## Provenance and peer review

Not commissioned, externally peer-reviewed.

## Declaration of competing interest

No potential conflict of interest relevant to this article was reported.

## Appendix A Supplementary data

Application Application
